# Therapeutical Intervention, Relaxation, Mental Images, and Spirituality (RIME) for Spiritual Pain in Terminal Patients. A Training Program

**DOI:** 10.1100/tsw.2006.345

**Published:** 2006-06-27

**Authors:** Ana Catarina de Araújo Elias, Joel Sales Giglio, Cibele Andrucioli de Mattos Pimenta, Linda Gentry El-Dash

**Affiliations:** ^1^ School of Medical Sciences, Universidade Estadual de Campinas (UNICAMP), Campinas (SP), Brazil; ^2^ Department of Medical Psychology and Psychiatry, School of Medical Sciences, Universidade Estadual de Campinas (UNICAMP), Campinas (SP), Brazil; ^3^ Department of Medical-Surgical Nursing, School of Nursing, Universidade de São Paulo (USP), São Paulo (SP), Brazil; ^4^ Department of Applied Linguistics, Universidade Estadual de Campinas (UNICAMP), Campinas (SP), Brazil

**Keywords:** alternative therapy, palliative care, spirituality, relaxation techniques, death, training program, near death experiences, Brazil

## Abstract

Therapeutic intervention involving the technique of Relaxation, Mental Images, and Spirituality (RIME) can foster the redefinition of spiritual pain in terminal patients. A training course was developed to instruct health care professionals in its use, and the results were followed up by evaluating reactions of professionals to its use in intervention with patients. Six subjects (a nurse, a doctor, three psychologists, and an alternative therapist), all skilled in palliative care, were invited to take part in the experience. They worked with 11 terminal patients in public hospitals of the cities of Campinas, Piracicaba, and São Paulo, located in Brazil. The theoretical basis for the study involves action research and phenomenology, and the results were analyzed using both qualitative and quantitative methods. The analysis of the experience of the professionals revealed 5 categories and 15 subcategories. The analysis of the nature of spiritual pain revealed 6 categories and 11 subcategories. The administration of RIME revealed statistically significant differences (*p* < 0.0001), i.e., patients reported a greater level of well-being at the end than at the beginning of sessions, which suggests that RIME led to the redefinition of spiritual pain for these terminal patients. The training program proposed has shown itself to be effective in preparing health care professionals for the use of RIME intervention.

> There is a Greater Love. There is a Greater Goodness. There is a Greater Power. Our mind is linked to the universe. We are not an isolated part of the universe. We are a part of it, along with all the other parts. We participate in the same respiration — the Great Respiration. The limited respiration of our lungs is only illusory. Our movement is illusory. Our real movement is mental, spiritual. All that we can see of everything that surrounds us depends on the level of our consciousness. (Celso Charuri)

## INTRODUCTION

Based on the elements cited by individuals who have undergone near death experiences (NDE)[1,2,3,4,5,6,7,8,9,10,11], the senior author developed a program of intervention for terminal patients to be administered by health care professionals[12,13,14,15,16,17,18], which provides a way of dealing with negative ideas about the meaning of life and death, and a redefinition of spirituality and the spiritual pain involved in fear of death or the afterlife and guilt before God. The program involves relaxation, mental images, and spirituality (RIME) with spirituality understood to constitute both the connection between the patient and the most transcendental area of the psyche[19] and the experience of greater love[20]. This paper reports on a course developed to train health care professionals to provide such therapeutic intervention for terminal patients.

Spirituality has become a growing issue in the medical field, as it was the subject of 1,222 studies cited in the international literature in health sciences (Medline) between 1993 and 2005, with 82 involving both spirituality and death, while only 2 articles were found for the 1966 to 1992 period. Prior to 2003, only 2 dealt with transcendence involving spirituality for critically ill patients. Barham[21] reported a case study using a Buddhist technique for experiencing the process of death, which promotes symptom control during the final 48 h of life. That author observed that aspects related to spirituality, serenity, peace, and love were very important for the sensation of security necessary for a “good” trip to the spiritual world.

Papathanassoglou and Patiraki[22] provided a hermeneutic and phenomenological perspective, focusing on the interpretation of dreams of individuals after hospitalization in an intensive care unit. The research explored the meaning of having been critically ill. Eight individuals reported their dreams in semi-structured interviews. Using the language of the unconscious, they were able to find the symbolic meaning of their experiences with critical illness conceptualized as a phase leading to transformations in the “self” by awakening spirituality and personal growth. According to the authors, nurses in an intensive care unit should be prepared to help patients experience the process described.

In a review of the literature involving intervention with terminally ill patients in the database of Medline for 1966–2005, the Latin-American and Caribbean Literature on Health sciences (LILACS), and the National Center for Biotechnology Information (NCBI) of the U.S. National Library of Medicine, various studies promoting intervention of some sort were found, but only a few specifically involved terminally-ill patients. Generally known as complementary or alternative therapies, these interventions involve the promotion of the quality of life during the process of dying through the use of music therapy[23], dignity therapy (which consists of caring for the patient focusing on his/her level of cognitive and functional independence and the control of physical and psychological symptoms[24], hypnosis[25]), and the application of religious or spiritual intervention (prayer and speech with a clergyman or meditation and directed imagination)[26]. Among the papers involving meditation and directed imagination is the work of Birnbaum and Birnbaum[27], who described an innovative therapeutic intervention in group work with survivors of suicide attempts and mental health professionals. The techniques used included relaxation and concentrated meditation, accompanied by directed meditation in a search for internal wisdom. Many participants related an important positive experience, including access to internal knowledge, which was highly relevant for them at that moment in their lives. For some, these introspections were felt to be coming from a deeper part of the patient’s own self (internal source), whereas for others, the source was felt to be a spiritual guide or presence (external source). The results indicated that directed meditation might be a powerful resource for therapists and their patients, including suicide attempts.

The methods used by many of these therapies resemble that of the RIME intervention, although the latter involves the specific induction of visualization of those elements described by patients who have passed through NDE. In this paper, we report on a course for training health care professionals in the use of RIME. Despite the growing importance, which health professionals have given to the question of spiritual assistance, training designed to minimize spiritual suffering has not previously been reported in Brazil.

Medical practitioners can no longer ignore alternative practices. We must recognize that certain alternative treatments may be more effective for a specific patient than the traditional allopathy, and such practices must be made available for use under appropriate circumstances. Curriculum planners must thus include such information during medical training. It will then be up to the health care professional to select the truly best possible options in his/her treatment of the population[28]. The training program of RIME intervention developed here may provide such an option for the curriculum and this preparation for dealing with terminally-ill patients could provide a contribution to the education of future health care professionals who will eventually have to work with such patients.

## METHODS

A training program was designed to prepare health professionals for the use of RIME intervention. The results were analyzed to explore the experience of these professionals in the utilization of this intervention and that of the patients in their redefinition of spiritual pain during the administration of RIME.

This is a study in action research and phenomenology. Phenomenological research[29] is generally defined as a study of what is lived and its meanings. The presupposition is that what is experienced is an important path to the truth and decisions that must be taken. It works on the level of intentions, and the analysis of this experience can be made in general (describe the nature of the experience) or specific (describe the meaning of specific experiences for a given individual or group of individuals), but it can also be concerned with the establishment of relationships of meaning. In this study, specific experiences were analyzed, i.e., the experiences of both patients and professionals applying RIME were investigated, as well as the nature of spiritual pain and its redefinition. According to Amatuzzi[29], there are various kinds of phenomenological research; the thesis presented in this article is based on empirical phenomenological research involving the application of phenomenology to psychological research based on empirical data. Action research, on the other hand, presupposes joint participation of the research worker and the subjects[30]; in this study, such collaboration involved the senior author and various health care professionals who were taught RIME so they could help transform the spiritual pain of terminal patients.

The qualitative results were subjected to content analysis involving semi-structured interviews, a structured questionnaire, and a diary. According to Bardin[31], content analysis can be conducted by the investigation of a specific theme (as well as by other techniques) and consists of separating a text into units, i.e., discovering "nuclei of meaning", which constitute the communication and which may, by their frequency, mean something in relation to the analytic objective involved. Afterwards, these affirmations (statements) are grouped in relation to a specific subject. The analysis can be presented graphically by means of words, phrases, or a summary. The quantitative results were analyzed by a descriptive method using data collected by the Analogical Visual Scale of Well-Being model with colored facial expressions. The scores at the beginning and end of each session were compared on the basis of averages, using a Wilcoxon test.

Six health care professionals participated in the training program and later administered RIME to terminal cancer patients; they included a nurse (responsible for the nursing team of the cancer sector of a university hospital specializing in the treatment of women), a physician (coordinator of the program for palliative care in a public hospital of the state of São Paulo in Brazil and president of the National

Academy of Palliative Care [ANCP]), three psychologists, and an alternative therapist. Two of the psychologists and the therapist work in the area of palliative care in the two hospitals mentioned above and the third psychologist (trained as a Jung analyst) had vast clinical experience and studied palliative care.

The patients were 11 individuals with terminal-stage cancer. They ranged in age from 27–76 years and had an educational level ranging from high school to a university degree. Their religions included Catholicism, various Protestant denominations, and Spiritism.

## The Program

A two-day preparatory course for RIME intervention was developed and administered by the first author during the first semester of 2004 in the public university in the city of Campinas in the state of São Paulo in Brazil. RIME intervention involved three stages. The first was the identification of symbolic pain of death, which was determined in a semi-structured interview. The second involved the transformation of the data about spiritual pain expressed in the interview into symbolic images, and the third consisted of orientation in the use of techniques of mental relaxation and the visualization of mental images to induce the elements that constitute spirituality. Complementary sessions for the orientation of family members are also recommended as part of the therapeutic intervention[12,13,14,15,16,17,18].

The pedagogic strategies used (expository classes, group discussions, and direct experiences) are included in the outline of the course presented in Table 1. The content information of the course was supplied in the form of a 100-page booklet.

## RESULTS

Data to evaluate the experience of health care professionals in the use of RIME methodology were collected from structured questionnaires, semi-structured interviews, and diaries. The analysis revealed 5 categories, with a total of 15 subcategories. The categories identified and the number of professionals making reference to that category, at least once, are presented in Table 2.

Analysis of the patient’s experience with spiritual pain and its redefinition identified 6 categories and 11 subcategories that referred to spiritual pain. These are presented in Table 3 along with the number of patients who reported each at least once.

The patients evaluated their well-being by means of the Analogic Visual Scale of Well-Being at the beginning and end of each RIME session. This scale involves evaluation on the basis of six colored faces expressing a range of suffering from none to unbearable. The data are presented in Table 4 and Fig, 1. The averages and medians were calculated for each patient, and the number of sessions in which there was an improvement is reported.

The difference in the scores before and after each session was based on the averages, using the Wilcoxon Test for nonparametric data. Statistically significant differences ($p < 0.0001$) were obtained, i.e., after RIME sessions, patients reported a greater level of well-being.

**TABLE 1**
**Outline of Course for Health Professionals for Therapeutic Intervention with Terminal Patients**

| Class | Contents | Duration | Strategy |
|---|---|---|---|
| Presentation of group | Reception, warm-up, and member introductions | 30 min | Experience |
| Presentation of training program | Structure of course and signing of informed consent | 30 min | Orientation, oral and multimedia presentation |
| Techniques of mental relaxation and visualization of mental images: theoretical aspects | Definition of mental relaxation; induction of mental relaxation; considerations of interaction between soma and psyche; definition of mental image visualization; short history of the utilization of image visualization in medicine; recent considerations in use of mental image visualization | 30 min | Oral and multimedia presentation |
| Experience (relaxation and visualization) | Projection of images of beautiful scenes; relaxation and visualization exercises based on images chosen | 30 min. | Oral and multimedia presentation |
| Theoretical foundations of spirituality adopted in the study | Presentation of theoretical background of program; reference to the meaning and dimensions of spirituality; NDE; spiritual needs of the terminally ill; dreams and experiences of a spiritual nature related to the terminal phase of illness | 30 min | Oral and multimedia presentation |
| Elements involved in the issue of spirituality (studies of NDE) | The spiritual world, death, and postdeath, as reported in NDE; description of elements commonly encountered when passing through NDE; identification of possible changes in (attitude?) after NDE; philosophical and spiritual considerations based on studies of NDE | 30 min | Oral and multimedia presentation |
| Documentary video | Report s of 6 patients about NDE | 60 min | Presentation of video |
| Dreams and experiences of spiritual nature related to terminal phase | Definition of experiences and dreams of spiritual nature; symbolic data of dreams and spiritual experiences of patients, family members, and the first author, collected during MSc thesis and interpreted based on Jungian references and elements of spirituality | 60 min | Oral and multimedia presentation |
| Synthesis of day | Identify main aspects brought up in classes of training program for the day | 60 min | Reflection and group conclusion |
| Therapeutic intervention: RIME | Main aspects of relationship management and therapeutic work; verbal and nonverbal symbolic language; structure of therapeutic intervention; family orientation | 90 min | Oral and multimedia presentation |
| Symbolic pain of death/ spiritual pain: case reports | Concepts of pain, total pain and spiritual pain; presentation of cases, including description of spiritual pain identified and operationalized during RIME | 90 min | Oral and multimedia presentation |
| Exercises of relaxation, visualization and spirituality | Exercise based on the main elements to be worked on with patients: beautiful scenes, symbols of transformation and beings of light; exchange of experiences of participants | 30 min | Verbal orientation for visualization and listening to music; report of experiences of group |
| Explanation of instruments used in the research | Objectives of the study; method and instruments for collection of data | 60 min | Presentation via multimedia |
| Summary of training | Summary of aspects covered in the group and sharing of experiences during the preparation course | 90 min | Group work of reflection and conclusion |

**TABLE 2**
**Summary of Categories Identified and Number of Professionals Referring to the Topic at Least Once**

| Category | No. of Professionals |
|---|---|
| Facilitation in managing therapeutic relationship | 6 |
| Awakening of feelings, perceptions, and emotions suggesting psychospiritual maturity in health professional | 6 |
| Feelings and experiences of spiritual and/or transcendental nature reflecting love and peace or the occurrence of intuitive dreams | 6 |
| Sometimes awakening of negative feelings and emotions in health care professionals | 4 |
| Perception of proposal as objective and feasible, leading to positive results in palliative care | 6 |

**TABLE 3**
**Summary of Categories and Subcategories Identified and Number of Patients Reporting Each at Least Once**

| Category | Subcategory | No. of Patients (n = 11) |
|---|---|---|
| Fear of death expressed by negation of gravity of clinical stage | Difficulty in accepting diagnosis and clinical care; attachment to concrete and material world | 5 |
| Fear of death expressed by perception of gravity of clinical stage | Concern with physical suffering; nonverbal expression of tension, fear, and panic; aggravation of clinical symptoms | 5 |
| Fear of after-death based on negative experience in spiritual dreams | Visualization of terrifying or very frightening images | 2 |
| Fear of after-death expressed as negative affective feelings of disintegration, nonexistence, or being forgotten | State of heightened alertness; anxiety of separation; doubts about Divine Love | 5 |
| Negative ideas and concepts about the meaning of life expressed by absence of feelings or feelings of existential emptiness | Previous negative experiences of affective or productive nature | 4 |
| Negative ideas and concepts of spirituality caused by experience of affective abandonment projected to spirituality | Difficulty in transcendence and trusting spiritual beings | 2 |

**TABLE 4**
**Well-Being Score of Patients (n = 8) using Visual Analogy Scale (VAS) Before and After RIME Intervention**

| Patient | No. of Sessions Evaluated | VAS Well-Being at Beginning of Session | VAS Well-Being at End of Session | No. of Sessions with Improvement |
|---|---|---|---|---|
| E.O.G | 3 | Score = 4+2+2<br>Total = 8<br>Mean = 2.6<br>Median = 2 | Score = 8+8+10<br>Total = 26<br>Mean = 8.6<br>Median = 8 | 3 |
| P.M. | 5 | Score = 0+0+0+0+0<br>Total = 0<br>Mean = 0<br>Median = 0 | Score = 4+4+8+4+0<br>Total = 20<br>Mean = 4<br>Median = 8 | 4 |
| M.S.S. | 4 | Score 4+2+0+2<br>Total = 8<br>Mean = 2<br>Median = 0 | Score = 6+6+6+10<br>Total = 28<br>Mean = 7<br>Median = 6 | 4 |
| N.J. | 3 | Score = 2+8+10<br>Total = 20<br>Mean = 6.6<br>Median = 8 | Score = 10+10+10<br>Total = 30<br>Mean = 10<br>Median = 10 | 2 |
| ZBO | 4 | Score = 5+5+6+6<br>Total = 22<br>Mean = 5.5<br>Median = 5.5 | Score = 5+5+6+7<br>Total = 23<br>Mean = 5.75<br>Median = 5.5 | 1 |
| M.L.C.I. | 1 | Score = 0<br>Total = 0<br>Mean = 0<br>Median = 0 | Score = 10<br>Total = 10<br>Mean = 10<br>Median = 10 | 1 |
| M.A.S. | 02 | Score = 6+2<br>Total = 8<br>Mean = 4<br>Median = 4 | Score = 8+8<br>Total = 16<br>Mean = 8<br>Median = 8 | 2 |
| M.V.C.S | 11 | Score =<br>6+6+6+6+6+6+6+6+6+6+6<br>Total = 66<br>Mean = 6<br>Median = 6 | Score =<br>10+10+10+10+10+10+10+10+10+10<br>Total =110<br>Mean = 10<br>Median = 10 | 11 |
| **Total** | **33** | **Total score = 132**<br>**Mean = 4**<br>**Median = 5** | **Total score = 263**<br>**Mean = 7.96**<br>**Median = 10** | **28** |

Wilcoxon Test: $p < 0.0001$.

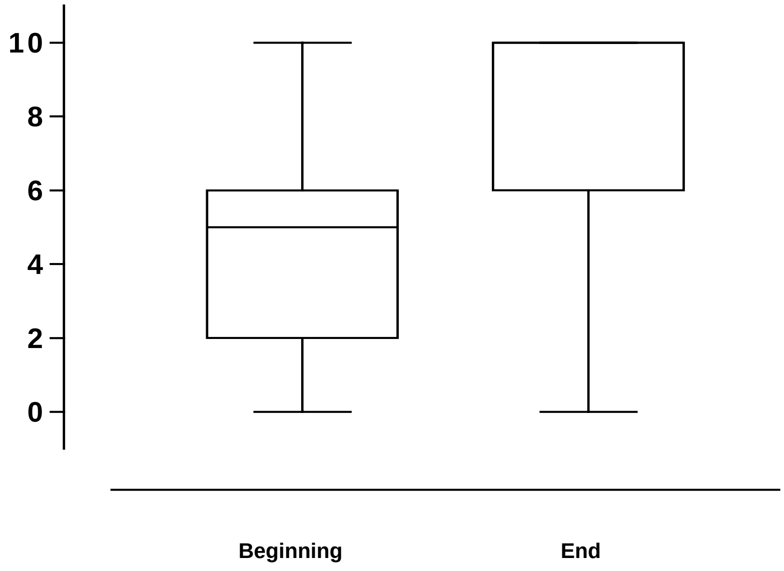

**PERCENTILE:**
**Beginning: 25% 2, 6**
**End: 75% 6, 10**
**MEDIANS:**
**Beginnings: 5**
**End: 10**

**FIGURE 1.** Blox plot scores of well-being reported by patients using VAS of Well-Being before and after RIME sessions.

## DISCUSSION

The professionals involved observed that the procedures of RIME (the attractive images of nature; music; visualization of places suggesting peace, tranquility, and harmony; and reference to spiritual beings transmitting love, goodness, serenity, and protection) favored the establishment of the relationship between professional and patient. The emotional climate in the therapeutic setting also favored the development of this relationship, since it favored the transcendental/spiritual representation of love and safety (through reference to the presence of beings of light, angels, etc).

Some of the reports made are cited here:

"I attribute the positive results to the therapeutic relationship established and the connection with beings of light. I felt like an angel carrying the patient." (Therapist)

"I felt the establishment of a relationship on the basis of the confidence and serenity expressed by the patients when they chose images and listened to music, and when they were involved in visualization. The family of one patient nicknamed me the "music girl"." (Psychologist)

"In one case, the elements for the administration of RIME had to be chosen based on information furnished by family members, because the patient could no longer communicate, but during the administration, the patient reacted positively, as I could see from his respiratory frequency and facial expressions." (Physician)

Therapeutic listening is an important instrument in the area of health; this can be effected by means of adequate professional intervention, as pointed out by Fiorini[32]. We have observed that the administration of individualized RIME, considering the personal history of the patient and his/her selection of music for relaxation, facilitated the establishment of therapeutic listening. This therapeutic listening then facilitated the context of visualization, as well as the specific images of nature, beings of light, and other symbols, which are personalized so that the definition of spiritual pain is unique for each patient.

The benefits of RIME accrued for both patients and professionals. There is a constant battle between the energy of life and death until the organism eventually gives up and is reduced to inorganic matter[33]. When the energy of love is present, however, the energy of life will be victorious as long as it has completed the construction of an action of fecundity, identified in the feelings, perception, and emotions referred to by the professionals. Here we cite relevant examples from the reports of the professionals.

"The only truth in the world is the manifestation of love. By administering RIME to a patient, I am transmitting love to that patient." (Psychologist)

"I felt good when I realized that I was actually helping someone at an extremely difficult time. I felt at peace when I administered RIME, and that peace extended to a peaceful resolution of other conflicts on the job." (Nurse)

"I felt transformed during the administration of RIME, since I could relax while helping my patient; moreover, each time I administer RIME, these good feelings return." (Psychologist)

RIME was found to be a feasible approach for the induction of the equivalent of NDE in terminal patients. Since the NDE is known to lead to a loss or reduction in the fear of death, it contributes to a serene and more dignified death, and such a benefit was also observed here. Moreover, not only the terminal patients, but also the professionals administering RIME, benefited from the experience, and the latter reported they had integrated the relevant aspects into their lives in a healthy and constructive way.

Based on the experiences reported by these professionals, including those of the senior author herself, we can affirm that during the administration of RIME, there is a psychic insertion of the professional into the limit between the physical world and the "spiritual world"; moreover, the "essence" or "spirit" of the professional and that of the patient become closer, or in psychoanalytic terms, there is an unconscious approximation of therapist and patient. We realize that RIME is not easy to administer; since it unites therapist and patient in a single feeling, it requires the commitment of the professional to this involvement.

As examples, we cite some of the statements of the professionals about the approximation involved:

"I felt an intuitive perception of the presence of beings of light during the application of RIME, and I felt a feeling of transcendence and relief in relation to my own spiritual questions." (Therapist)

"I felt a sense of peace and tranquility when I reflected on the impending death of my patient and the way in which this would be less traumatic for her and her family; when I left the session, I felt a clear feeling of love and peace and relief. These sensations were very strange, since they emanated from beings of light in the environment."(Psychologist)

"I felt renovated after applying RIME, although normally I would have been exhausted. I also had an intuitive perception of the presence of beings of light in the environment, both during and after the application of RIME, and I felt safe and protected in this presence."(Physician)

We also observed that some of the professionals reported that some patients were extremely afraid of death at the beginning of the sessions. When these professionals heard about their spiritual pain, they felt similar sensations themselves and reported feelings of impotence and frustration in the face of the resistance of their patients. Since RIME involves therapist and patient in a single relationship of unconscious approximation, these feelings are understandable. Once the elements of RIME have been experienced, these negative emotions disappear. As an example, we cite a statement of one of the psychologists: "At the beginning of the sessions with my second patient, I was unable to sleep. I was extremely worried about death and had nightmares. I think these feelings were probably due to my contact with the energy evolving from my patient's fear of death."

All of the professionals considered RIME to be a feasible intervention procedure that provides positive results in the treatment of patients in the stage of palliative care. Examples of some of their affirmations follow:

"The application of RIME contributed to my personal and professional development and left me with a more complete position in both spheres. And a feeling of safety and serenity in the face of the dichotomy between death and life. I learned not to feel prejudice, when I felt compassion/sympathy." (Psychologist)

"I was involved with a struggle between the real and the spiritual, and RIME serves an opening for a solution in this struggle. The inclusion of the issue of spirituality in conventional medical care does not interfere with clinical treatment and the patient's trust of the doctor, but rather strengthens it. After learning about and applying RIME, I can no longer be satisfied by just caring for the physical needs of a terminal patient." (Physician)

"RIME brought serenity and trust to the patients, and this was perceived both verbally and nonverbally by their family members. After this experience, my professional actions extend beyond physical care to include the fostering of psychic and spiritual relief, as well as eventual peace for the patient."(Nurse)

Nine of the 11 patients revealed a fear of death with this aspect of spiritual pain being characterized as the most prevalent and responsible for most of their suffering during the terminal process; this is in agreement with the results of previous studies[13]. One of the patients in an intermediate stage of negation/perception was oscillating between the two positions, but revealed aspects of the fear of death in both. Seven of the patients, including the two who did not mention a fear of death itself, reported fear of the afterlife. It seems that an understanding of such representations of spiritual pain is very important, since professionals, especially nursing technicians, often take the attitudes of patients personally, which generates unnecessary professional stress, as well as contributing to inadequate assistance in meeting the needs of the patient.

In both qualitative analysis and quantitative analyses, RIME intervention facilitated the redefinition of spiritual pain. The quantitative analysis revealed a statistically significant difference ($p < 0.0001$), between the level of well-being at the beginning of sessions and at the end. The redefinition took place gradually over time, although these feelings worsened during the interval between sessions. It is thus recommended that the interval between sessions be as brief as feasible. RIME intervention minimized the suffering during the process of dying for a diversified population of patients with some sort of cancer. Patients of both genders with educational level ranging from a minimum of formal education to a university degree were treated successfully, nor did age seem to make a difference, as RIME was effective for people ranging in age from 27–76 years and during the pilot project we also worked with children and teenagers, also with good results[14]. Moreover, the effectiveness was not compromised by religious beliefs, as it was successful with Catholics, Spiritists, and various Protestant sects,

In fact, we did not find any counter-indications for the administration of RIME during any of the phases of our study, although it is not for everyone. Its administration should probably be limited to health care professionals and patients who believe in a spiritual life of some sort, independent of their religion. One of the professionals participating in the initial phase of instruction was a nurse who found herself unable to administer the technique with terminal patients, even though she was quite experienced in the area of palliative care (and works in the home care services of a public hospital). An interview revealed that she was unable to deal with the criticism of other professionals who thought that she should be concerned only with the biological aspects of her patients, rather than their psychological and spiritual aspects. We feel that these observations are pertinent; since administration of RIME intervention may require special courage on the part of professionals in order to confront the resistance that may be encountered during this period of paradigm shift[34].

## CONCLUSIONS

The program reported here proved to be effective for the training of health care professionals (nurses, physicians, psychologists, and therapists) to administer RIME, and this treatment increased the well-being of terminal patients by improving the quality of their lives during the process of dying, as well as facilitating the redefinition of their spiritual pain. The result of this intervention was greater serenity and more dignity in the face of death, and the reactions of both professionals and patients to the use of RIME

was overwhelmingly positive. Limitations of this study include the fact that the research design did not include the utilization of a control group to compare the results of RIME with other types of intervention, although in the future we intend to include a control group. Moreover, the sample was too small to permit generalization. Although the results reached significance and were collected using a rigorous academic methodology, the number of patients and professionals involved was minimal. New studies involving more individuals are called for to develop a better understanding of the experience of professionals administering RIME intervention, especially the spiritual aspects of this experience, as well as that of redefining the spiritual pain of patients.

This study involved only patients with terminal cancer, but RIME should be helpful in other situations such as pre- and postsurgery, the diagnosis and accompaniment of cancer, and the treatment of psychosomatic illnesses, as well as the therapy of patients who have attempted suicide. Its beneficial effects for the treatment of health care professionals should also be investigated.

## BIOSKETCHES

**Ana Catarina de Araújo Elias, PhD**, Psychologist, School of Medical Sciences, Universidade Estadual de Campinas (UNICAMP), Campinas (SP), Brazil. Professor of Psychology of the School of Biomedical Sciences of the Centro Universitário Nossa Senhora do Patrocínio (CEUNSP), Itu (SP), Brazil. anacatarinaelias@uol.com.br or acatarina@fcm.unicamp.br

**Joel Sales Giglio, PhD**, Psychiatrist. Associate Professor of the Department of Medical Psychology and Psychiatry, School of Medical Sciences, Universidade Estadual de Campinas (UNICAMP), Campinas (SP), Brazil. Jungian Analyst by the Jungian Association of Brazil. Education director of the Jungian Association of Brazil (AJB).

**Cibele Andrucioli de Mattos Pimenta, PhD**, Full (Titular) Professor of the Department of Medical-Surgical Nursing, School of Nursing, Universidade de São Paulo (USP), São Paulo (SP), Brazil.

**Linda Gentry El-Dash, PhD**, Associate Professor of the Department of Applied Linguistics, Institute of Language Studies, Universidade Estadual de Campinas (UNICAMP), Campinas (SP), Brazil.
